# Molecular Features of Classic Retinal Drugs, Retinal Therapeutic Targets and Emerging Treatments

**DOI:** 10.3390/pharmaceutics13071102

**Published:** 2021-07-20

**Authors:** Alessandro Arrigo, Francesco Bandello

**Affiliations:** IRCCS San Raffaele Scientific Institute, Vita-Salute San Raffaele University, Via Olgettina 60, 20132 Milan, Italy; alessandro.arrigo@hotmail.com

**Keywords:** retinal diseases, anti-VEGF, corticosteroids, intravitreal injections, complement inhibitors, chemokine receptor inhibitors, integrins inhibitors, tyrosine kinase inhibitors, nutraceutics

## Abstract

The management of exudative retinal diseases underwent a revolution due to the introduction of intravitreal treatments. There are two main classes of intravitreal drugs, namely anti-vascular endothelial growth factors (anti-VEGF) and corticosteroids molecules. The clinical course and the outcome of retinal diseases radically changed thanks to the efficacy of these molecules in determining the regression of the exudation and the restoration of the macular profile. In this review, we described the molecular features of classic retinal drugs, highlighting the main therapeutic targets, and we provided an overview of new emerging molecules. We performed a systematic review of the current literature available in the MEDLINE library, focusing on current intravitreal molecules and on new emerging therapies. The anti-VEGF molecules include Bevacizumab, Pegaptanib, Ranibizumab, Aflibercept, Conbercept, Brolucizumab, Abicipar-pegol and Faricimab. The corticosteroids approach is mainly based on the employment of triamcinolone acetonide, dexamethasone and fluocinolone acetonide molecules. Many clinical trials and real-life reports demonstrated their efficacy in exudative retinal diseases, highlighting differences in terms of molecular targeting and pharmacologic profiles. Furthermore, several new molecules are currently under investigation. Intravitreal drugs focus their activity on a wide range of therapeutic targets and are safe and efficacy in managing retinal diseases.

## 1. Introduction

The human retina may be affected by two macro groups of diseases, namely maculopathies and retinopathies. Whereas maculopathies are confined to the central part of the retina, bounded by the vascular arcades, retinopathies may extend up to the extreme retinal periphery. These two categories can be further subdivided according to the main features characterizing the disease, thus taking into consideration exudative or atrophic phenomena.

Exudation is an active process, and its nature depends on each specific retinal disease, causing fluid to accumulate within the retina or in the subretinal space. It mainly involves variable amounts of fluid, the major pathogenic features of which are the breakdown of the blood-retinal barrier and increased inflammation [1,2,3]. Retinal diseases can also be characterized by other types of debris, including lipofuscin and lipidic and proteinaceous materials [3,4]. Retinal diseases can also be characterized by the progressive degeneration of inner and outer retinal layers. These atrophic changes may occur independently or in the context of an initial exudative disease [3,5].

Current retinal therapeutic approaches are based on these premises and designed to prompt the exudation to regress, stimulate debris reabsorption or prevent the atrophy from expanding. In this review, we discuss the biochemical properties of the main retinal drugs, focusing on the association between their specific features and their therapeutic employment in retinal diseases.

## 2. Methods

We used keywords to explore all English language human subject articles in the MEDLINE library. The keywords included the following: retinal disease, exudation, atrophy, diabetic retinopathy, diabetic macular edema, age-related macular degeneration, geographic atrophy, retinal vein occlusion, retinal dystrophy, vascular endothelial growth factor, VEGF, anti-VEGF, intravitreal injections, steroids, corticosteroids, dexamethasone implant, DEX implant, fluocinolone acetonide implant, emerging treatment, complement inhibitor, integrin inhibitor. All the references were carefully examined by two expert researchers (FB, AA), who collated and arranged all the relevant information, bearing in mind this review’s main theme as expressed in the manuscript title.

## 3. Retinal Drugs for Exudative Diseases

The prognosis of retinal exudative diseases changed radically after the introduction of intravitreal therapies. While the old laser-based treatments were effective in blocking exudation, they were associated with an extremely poor visual outcome [6,7,8]; nowadays, patients can expect to preserve their quality of life and a good visual function. The current intravitreal therapeutic bullets consist of anti-vascular endothelial growth factor (anti-VEGF) and corticosteroids. The pros of anti-VEGF drugs are their easier management and the low instance of side effects; the cons comprise their limited duration, meaning a large number of injections are required, and their contraindication in patients displaying a high risk of cardiovascular dysfunction. In contrast, the pros of corticosteroids include their longer duration, thus reducing the number of injections administered and their greater anti-inflammatory action. Conversely, corticosteroids are closely associated with an increase in intraocular pressure and a faster progression of cataracts.

In this review, we discuss the following anti-VEGF molecules: Bevacizumab (Avastin^®^, Hoffmann-La Roche, Basel, Switzerland), Pegaptanib (Macugen, Eyetech/Pfizer, New York, NY, USA), Ranibizumab (Lucentis^®^, Novartis Pharmaceuticals, Ottawa, Canada), Aflibercept (Eylea^®^, BAYER Pharma AG, Leverkusen, Germany), Conbercept (Chengdu Kanghong Biotech Company, Sichuan, China), Brolucizumab (Beovu^®^, Novartis Pharmaceuticals, Ottawa, ON, Canada), Abicipar-pegol (Allergan, Inc., Dublin, Ireland) and Faricimab (Hoffmann-La Roche, Basel, Switzerland). We also examine the biochemical properties of the following corticosteroids: triamcinolone acetonide, dexamethasone (DEX) (Ozurdex^®^, Allergan, Inc., Irvine, CA, USA) and fluocinolone acetonide (FAc) (Iluvien^®^, Alimera Sciences, Inc., Alpharetta, GA, USA). We go on to assess emerging retinal disease therapies, such as complement inhibitors, integrin inhibitors and the new generation of molecules.

## 4. Vascular Endothelial Growth Factor in the Human Retina

Since most intravitreal molecules have been developed to act as a blockage of vascular endothelial growth factor (VEGF), we found it useful to provide a brief overview of the VEGF properties in the human retina. VEGF is a dimeric glycoprotein of ~40 kDa and is fundamental in promoting angiogenesis during the development of the retina in vertebrates [9,10]. VEGF is actually a family of proteins that includes VEGF-A, VEGF-B, VEGF-C, VEGF-D, VEGF-E, VEGF-F and PGF (placental growth factor). These originate from the splicing of a source molecule and are further characterized by different isoforms [11]. All VEGF forms bind to different types of VEGF tyrosine kinase trans-membrane receptors; VEGFR-1/Flt-1 (fms-like tyrosine kinase) and VEGFR-2/KDR/Flk-1 (kinase insert domain containing receptor/fetal liver kinase) are mainly associated with angiogenesis [12], whereas Flt-3/Flk-2 and VEGFR-3/Flt-4 are involved in hematopoiesis and lymphangiogenesis [13]. VEGF production is up-regulated by the different isoforms of hypoxia inducible factor-1 (HIF-1) to promote physiological angiogenesis, whereas other HIF isoforms are involved in the regulation of VEGF expression [14]. Interestingly, VEGF production can also be increased by insulin-like growth factor 1 (IGF-1), playing an important role in retinal angiogenesis [15]. The main sources of VEGF are retinal pigmented epithelium (RPE) cells [16], astrocytes [17], Müller cells [18], endothelium and ganglion cells [19]. In pathological conditions, VEGF production is upregulated by increased hypoxia and oxidative stress [20,21]; however, other mediators are involved in the stimulation of VEGF release. Indeed, these mediators were found to have increased in retinal exudative diseases, including erythropoietin (EPO) [22], angiopoietins 1 and 2 (Ang-1 and Ang-2), along with the Tie2 receptor [12] and platelet-derived growth factor (PDGF) [23]. VEGF is the chief cause of exudative and neovascular phenomena; its increased level is also behind the formation of drusen in age-related macular degeneration (AMD), understood as extremely complex debris consisting of cholesterol, apolipoproteins, oxidated lipoproteins, glycoproteins, crystallins, components of the extracellular matrix and serum albumin [24]. Neovascularization and exudation processes are also mediated by the dysregulation of the VEGF/pigment epithelium-derived factor (PEDF) ratio. PEDF is a major inhibitor of angiogenesis [25] and it is more fully expressed in the peripheral RPE than in macular RPE [26], thus providing the basis of an explanation as to why neovascularizations and macular edema mainly occur in the macular region.

## 5. Anti-VEGF Molecules

Anti-VEGF are antibody molecules developed to bind VEGF and avoid the interaction with its receptors.

### 5.1. Bevacizumab

Bevacizumab (Avastin^®^, Hoffmann-La Roche, Basel, Switzerland) is a fully humanized IgG1 molecule binding VEGF-A isoforms. This antibody of 148  kDa was developed for cancer therapy [27] and it is currently employed as intravitreal “off-label” treatment for retinal diseases [28]. Bevacizumab’s mechanisms of function are quite simple; as a pure anti-VEGF antibody, its main effect is to block the neovascular stimulus and VEGF-induced vascular permeability [29]. Furthermore, bevacizumab interacts with HIF-1, reducing its stimulating effect on VEGF production [30]. Although several studies have described bevacizumab as an efficient and cost-effective treatment for retinal diseases [31,32,33], its usage is partially limited by its “off-label” classification.

### 5.2. Pegaptanib

Pegaptanib (Macugen, Eyetech/Pfizer, New York, NY, USA) was the first drug to obtain FDA approval for intravitreal administration. It is a pegylated-aptamer that binds preferentially to the heparin-binding domain of VEGF165 isoform [34]. Although proving to be efficient in inhibiting the neovascularization process [35], its molecular features strongly limit its capacity to block VEGF-related pathways, thus making it a subsidiary therapeutic choice.

### 5.3. Ranibizumab

Ranibizumab (Lucentis^®^, Novartis Pharmaceuticals, Ottawa, ON, Canada) is a recombinant humanized immunoglobulin G1κ isotype monoclonal antibody fragment (Fab) of 48 kDa binding VEGF-A isoforms and avoiding the interaction with VEGFR1 and VEGFR2. The lack of fragment crystallizable (Fc) domain and small molecule size might expand its affinity for more isoforms of VEGF-A (VEGF165, VEGF121, and VEGF110) and increase the diffusion of the molecule within the retina and choroid [36,37]. Furthermore, since ranibizumab shows only one binding site for VEGF, two molecules of ranibizumab bind to one VEGF dimer [38], with the ranibizumab/VEGF-A complex having a higher stability energy than bevacizumab [39] and greater molecular affinity than bevacizumab and aflibercept [40].

### 5.4. Aflibercept

Aflibercept (Eylea^®^, BAYER Pharma AG, Leverkusen, Germany) is a dimeric glycoprotein of 115 kDa, also known as VEGF Trap, a molecule obtained from the fusion of the first three Ig domains of VEGFR1 and the Fc region of human IgG1 [41]. These biochemical properties ensure aflibercept has a high affinity for VEGF-A isoforms and PIGF, as well as a relative affinity for VEGF-B. Ziv-aflibercept (Zaltrap; Sanofi-Aventis and Regeneron Pharmaceuticals, Inc, Tarrytown, NY, USA), a molecule differing from aflibercept only in its excipients and higher osmolarity, displays an almost identical biochemical profile [42]. In particular, if compared to the vitreous, aflibercept is iso-osmolar, whereas Ziv-aflibercept is hyperosmolar [42]. Although Ziv-aflibercept has been associated with promising effects in macular diseases, its usage is still off-label [43].

### 5.5. Conbercept

Conbercept (Chengdu Kanghong Biotech Company, Sichuan, China) is currently the third most popular molecule belonging to VEGF Trap family. It consists of a full human DNA sequence of 143 kDa, characterized by the fusion of extracellular domain 2 of VEGFR1 and extracellular domains 3 and 4 of VEGFR2 with the Fc portion of human IgG1 [44,45]. The pharmacokinetic profile of conbercept is quite similar to aflibercept’s; the main difference regards the presence of a portion dedicated to VEGFR2, which was developed to potentially increase the efficacy and stability of conbercept and to produce relative affinity for VEGF-C [44,45].

### 5.6. Brolucizumab

Brolucizumab (Beovu^®^, Novartis Pharmaceuticals, Ottawa, ON, Canada) is a single-chain antibody fragment of 26 kDa, characterized by the absence of the Fc portion and specifically developed to minimize molecule size and to improve the affinity for VEGF-A isoforms, compared with the other molecules [46,47]. Brolucizumab has been recently approved for use in neovascular age-related macular degeneration, showing non-inferiority and higher penetrance within the retina and the choroid compared with the other anti-VEGF molecules [48,49].

### 5.7. Abicipar-Pegol

Abicipar-pegol (Allergan, Inc., Dublin, Ireland) belongs to the family of designed ankyrin repeat proteins (DARPins) molecules, a class of molecules that can mimic antibodies and show a high affinity for the VEGF target [50]. More specifically, Abicipar-pegol is a recombinant protein of 34 kDa coupled to a polyethylene glycol fraction binding all VEGF-A isoforms [51]. Its affinity for VEGF-A turned out to be comparable to aflibercept’s but remarkably greater than bevacizumab’s and ranibizumab’s [52].

### 5.8. Faricimab

Faricimab (Hoffmann-La Roche, Basel, Switzerland) is the second molecule belonging to the DARPin class. The feature of this 150 kDa molecule is that it has two different targets; indeed, it can simultaneously and independently bind and neutralize both VEGF-A and Ang-2, the latter enabling interference with the Ang-1/Tie2 pathway to occur [53]. As mentioned above, the Ang-1/Tie2 pathway is a major pathogenic factor in the development of neovascularization and exudation. Bearing this in mind, Faricimab offers an interesting multitargets approach.

### 5.9. Clinical Remarks on Anti-VEGF

**Figure 1:**
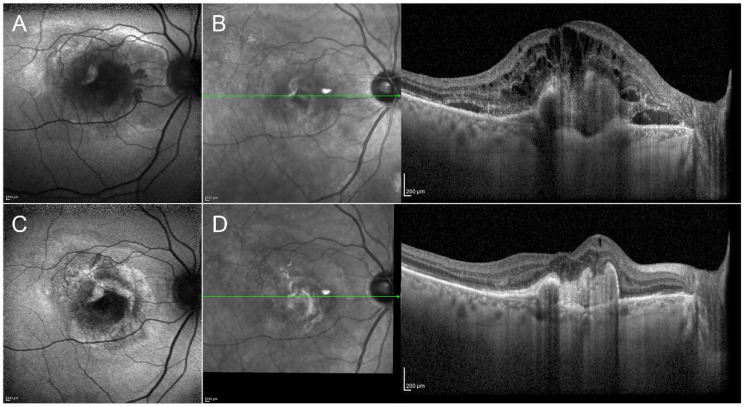
Clinical efficacy of anti-VEGF injections in macular neovascularization secondary to age-related macular degeneration. The onset of the neovascularization is characterized by evident changes of the fundus autofluorescence signal (**A**). Structural OCT describes a completely altered macular profile, with the presence of wide subretinal and intraretinal exudation (**B**). After the loading dose of three-monthly intravitreal injections of anti-VEGF, it is possible to observe an improvement of the fundus autofluorescence profile (**C**), together with the complete regression of the exudation detected by structural OCT (**D**).

There is a general consensus regarding the efficacy and safety of anti-VEGF intravitreal injections in macular exudative and neovascular diseases (Figure 1) [54,55,56,57,58]. Owing to their relatively simple management and good tolerance, bevacizumab, ranibizumab and aflibercept are often used as a first line strategy. The main disadvantages regard the contraindication for patients at high cardiovascular risk and the large number of injections required, thus placing a considerable burden on public health systems. Brolucizumab recently obtained FDA approval for use in neovascular age-related macular degeneration [59] and Faricimab is due to obtain similar approval [60,61]; both drugs are currently being tested for other retinal diseases. All the other cited molecules are currently under investigation through multicenter clinical trials.

## 6. The Role of Inflammation in the Human Retina

All retinal diseases are characterized by pro-inflammatory alterations, differing according to the specific pathogenic features. A comprehensive discussion of all the etiopathological features typical of retinal inflammation would be extremely involved and beyond the scope of the present review. For this reason, we intend to provide just a few useful gobbets to better understand the therapeutic role of corticosteroids in retinal diseases.

Inflammation may derive from progressive accumulation of pathologic debris, such as in the first stages of age-related macular degeneration; in these cases, pro-inflammatory mediators are stimulated by progressive and chronic increases of oxidative stress and cytotoxicity produced by these accumulations [24]. Furthermore, the progressive degeneration of retinal cells may stimulate complement activation and lead to the accumulation of microglia and inflammatory cells, and the onset and progression of pro-inflammatory phenomena [62]. At the same time, some retinal diseases reveal major pro-inflammatory sources. Inflammation is a key component of diabetes mellitus and diabetic retinopathy; progressive metabolic dysfunctions leading to increasing levels of advanced glycation end products and free radicals causes a chronic pro-inflammatory status, with progressive increases of pro-inflammatory cytokines, chemokines, and other inflammatory mediators, the accumulation of inflammatory cells and increases in the vascular permeability [63,64]. High pro-inflammatory mediator release is also a major phenomenon found in another frequent retinal disorder, namely retinal vein occlusion, where increasing levels of pro-inflammatory cytokines, interleukins and other factors have been encountered [65]. Inflammation is the key mechanism in uveitis [66] and is also involved in the pathogenesis of retinal dystrophies [67].

## 7. Corticosteroids

Intravitreal corticosteroids are employed to address the pro-inflammatory cascade characterizing several retinal diseases. Owing to their low water solubility, sustained-release delivery systems are needed to guarantee the therapeutic effect within the eye.

### 7.1. Triamcinolone Acetonide

Triamcinolone acetonide is a more efficient, salt form of triamcinolone, with a molecular weight of 434.5 g/mol. It displays a high affinity for cytosolic glucocorticoid receptors [68]. By interfering with the inflammatory cascade, triamcinolone acetonide can induce the regression of the exudation typical of both inflammatory and non-inflammatory retinal diseases. It can be administered through the following routes: peribulbar, sub-tenon, sub-conjunctival, intravitreal, retrobulbar and suprachoroidal [69]. Triamcinolone acetonide mainly acts by reducing the expression of endothelial adhesion molecules [70], while also exhibiting a moderate anti-vascular effect by inhibiting the basic fibroblast growth factor [71]. However, the fact that it was not specifically developed for use in the eye, combined with a high instance of toxicity phenomena [72], makes triamcinolone acetonide a secondary choice.

### 7.2. Dexamethasone

DEX implant (Ozurdex^®^, Allergan, Inc., Irvine, CA, USA) is an eye-specific intravitreal formulation developed to provide up to four months of treatment duration. DEX, which has a molecular weight of 392.5 g/mol, is a synthetic adrenal corticosteroid that displays a high affinity for nuclear glucocorticoid receptors and interferes with NF-kB activation and apoptosis [73]. The intravitreal concentration reaches its peak after 60 days, although DEX release from the soluble implant is not constant over the entire window of duration [74]. DEX exerts pleiotropic effects on pro-inflammatory mediators, acting as a powerful anti-inflammatory drug and as a VEGF inhibitor [74].

### 7.3. Fluocinolone Acetonide

FAc implant (Iluvien^®^, Alimera Sciences, Inc., Alpharetta, GA, USA) is the most recent corticosteroid to be approved for the management of diabetic macular edema [75]. FAc, whose molecular weight is 452.5 g/mol, is the acetonide salt form of fluocinolone and acts to bind cytosolic glucocorticoid receptors and subsequently translocated within the nucleus [76]. FAc performs its main anti-inflammatory action by inhibiting the synthesis of prostaglandins and leukotrienes, while also acting as a vasoconstrictor by inhibiting nitric oxide production [76]. In addition to the 0.19 mg non-biodegradable 25-gauge implant (Iluvien^®^, Alimera Sciences, Inc., Alpharetta, GA, USA), a 0.59 mg non-biodegradable pars plana sutured implant (Retisert, Bausch & Lomb, Bridgewater, NJ, USA) is also available. Iluvien^®^ technology ensures a daily release of 0.2 µg at a constant rate over three years; FAc concentration is at its highest during the first months after the implant and tends to decrease gradually until it reaches stability at around the sixth month, maintained thereafter until the end of the treatment [77,78]. FAc implantation is currently under investigation as a possible option for the long-term management of uveitis [79].

### 7.4. Clinical Remarks on Corticosteroids

**Figure 2:**
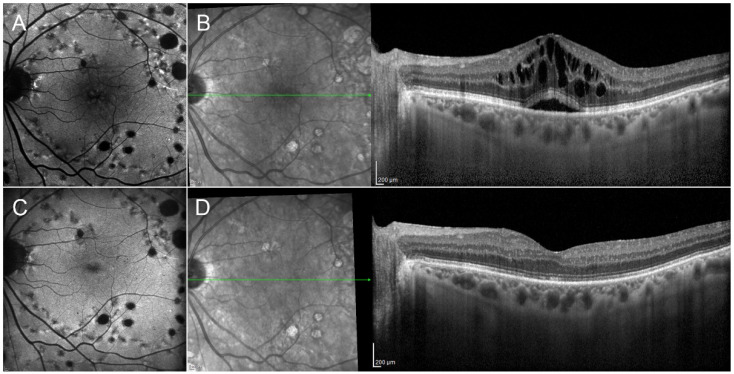
Clinical efficacy of dexamethasone implant in diabetic macular edema. The reported case has been already treated by laser, as shown by the hypoautofluorescent multiple spots (**A**), and it shows an evident macular edema with also the presence of subretinal fluid, detected on structural OCT (**B**). After a single dexamethasone implant, it is possible to observe the regression of the diabetic macular edema, with the complete recovery of the foveal profile (**C**,**D**).

Intravitreal implants of corticosteroids are valid therapeutic options for the management of retinal diseases involving chronic inflammation (Figure 2) [80,81,82]. These drugs are often considered as a second choice in retinal vascular diseases, unlike in the case of uveitis, where corticosteroids are a primary choice. Among the main disadvantages of employing intravitreal corticosteroids are the fast progression of cataract and the high risk of intraocular pressure (IOP) increases and glaucoma. The etiology of steroid-induced cataract is extremely complex and involves corticosteroids interference at different levels of lens homeostasis, including altered gene transcription, protein degeneration and changes to lens epithelial cell metabolism [83]. Although representing a vision threatening complication, it can be easily managed through cataract surgery. Of these corticosteroids-related complications, the most damaging is the increase in IOP. The pathogenesis of steroid-induced glaucoma is multifactorial and only partially understood. It includes reduced activity of the trabecular meshwork and the accumulation of extracellular debris, leading to reduced aqueous humor outflow and the development of a genetic predisposition [84,85,86]. The impact of IOP increases in clinical practice is high, since at least 20–30% of cases treated by intravitreal corticosteroids require IOP-lowering medications, and at least 4% of cases undergo IOP-lowering surgery. For these reasons, intravitreal corticosteroid treatment requires careful patient selection and monitoring, although it remains a useful option.

## 8. Emerging Therapies for Exudative Retinal Diseases

**Figure 3:**
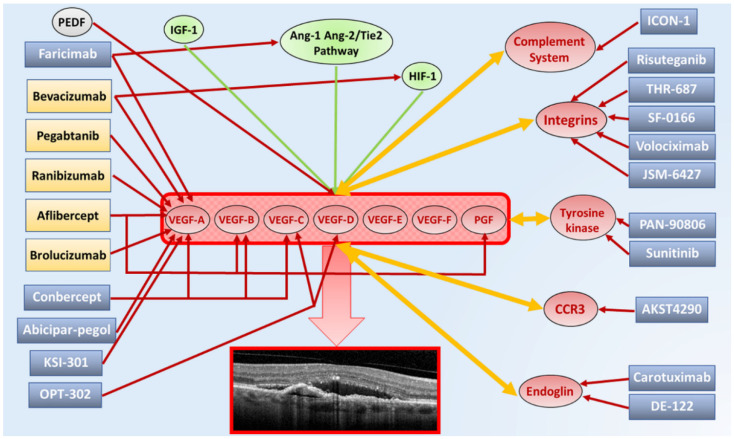
Summary diagram showing present and experimental molecular targets and corresponding intravitreal drugs for exudative age-related macular degeneration (AMD). The upregulation of vascular endothelial grow factor (VEGF) family expression, made by VEGF-A, VEGF-B, VEGF-C, VEGF-D, VEGF-E, VEGF-F and PGF, is responsible for the onset and progression of macular neovascularization and fluid production in exudative AMD. All VEGF molecule subtypes bind to different types of VEGF tyrosine kinase trans-membrane receptors. VEGF family production and release is further stimulated by increased levels of angiotensin 1-2 (Ang-1-Ang-2)/Tie2 pathway, insulin-like growth factor 1 (IGF-1) and hypoxia inducible factor-1 (HIF-1) (green arrows). Furthermore, a natural VEGF inhibitor is represented by PEDF (red arrow), resulting dysregulated in exudative AMD. Currently, approved anti-VEGF molecules include bevacizumab, pegaptanib, ranibizumab, aflibercept and brolucizumab. Although most of current anti-VEGF drugs mainly bind VEGF-A isoforms (red arrows), bevacizumab shows mild affinity also for HIF-1, while aflibercept also binds PGF. Anti-VEGF molecules under investigation include faricimab (VEGF-A isoforms + Ang-1-Ang-2/Tie2 pathway), conbercept (VEGF-A + VEGF-B + VEGF-C isoforms), abicipar-pegol (VEGF-A isoforms), KSI-301 (VEGF-A isoforms) and OPT-302 (VEGF-C + VEGF-D isoforms). Exudative AMD is also characterized by upregulation of complement system, integrins, tyrosine kinase, eotaxin C-C chemokine receptor type 3 (CCR3) and endoglin, which contribute to the increased production and release of VEGF family and often are themselves stimulated by the increased level of VEGF isoforms (orange arrows). Several molecules are currently under investigation through multicenter clinical trials, targeting these cofactors of the neovascular process, including complement system inhibitors (ICON-1), integrins inhibitors (risuteganib, THR-687, SF-0166, volociximab, JSM-6427), tyrosine kinase inhibitors (PAN-0806, sunitinib), CCR3 inhibitors (AKST4290) and endoglin inhibitors (carotuximab, DE-122).

In this section, we provided a short description regarding the molecules currently under investigation in multicenter clinical trials. A comprehensive scenario regarding the present and future intravitreal drugs for exudative retinal diseases, with particular regards to exudative AMD, are reported in Figure 3.

### 8.1. AKST4290

AKST4290 (Alkahest, San Carlos, CA, USA) is the inhibitor of the natural receptor for eotaxin C-C chemokine receptor type 3 (CCR3), a molecule proving to be highly expressed in choroidal neovascularization [87]. This molecule is under investigation as an oral formulation (400 mg), combined with intravitreal anti-VEGF injections, in AKST4290–201 (NCT03558061) and AKST4290–202 (NCT03558074) phase 2a clinical trials.

### 8.2. Carotuximab

Carotuximab (DE-122) (SANTEN, Osaka, Japan; TRACON Pharmaceuticals, San Diego, CA, USA) is an antibody directed against endoglin, and was developed on the basis of evidence showing endoglin to be actively involved in angiogenesis [88]. It has been found that a hypoxic status may stimulate the production of endoglin, which is also enhanced in actively proliferating endothelial cells. A mouse model of neovascularization has provided promising results regarding the use of anti-endoglin in combination with anti-VEGF injections [89]. DE-122 is under investigation in a phase 2a randomized controlled trial (NCT03211234).

### 8.3. Complement Inhibitors

The complement system is made up of an extremely complex network of molecules that play a fundamental role in innate immunity and in the activation of the inflammatory cascade. The role of the complement system has been widely demonstrated in both exudative and dry retinal diseases; complement activation is a major factor in enhancing pro-inflammatory mechanisms and in the onset and progression of cell death [90,91,92]. For these reasons, complement inhibitors are currently under investigation for both forms of retinal diseases, through the development of different molecules acting at multiple levels in the complement system’s activation cascade. The evidence regarding the efficacy of complement inhibitor drugs is not conclusive as yet, and there are many ongoing clinical trials designed to demonstrate the rationale for employing these molecules in wet and dry retinal diseases [93,94,95].

### 8.4. ICON-1

ICON-1 (Iconic Therapeutics, San Francisco, CA, USA) is an anti-tissue factor immunoconjugate protein produced by linking recombinant modified factor VIIIa protein with the Fc portion of a human IgG1. This molecule has been developed to counter the overexpression of anti-tissue factor by choroidal neovascularization. It has been separately tested in neovascular age-related macular degeneration patients, in combination with ranibizumab (EMERGE phase 2 clinical trial) [96] and with aflibercept (DECO phase 2 clinical trial) (NCT03452527), showing further improvement of neovascularization regression, compared with anti-VEGF injections alone.

### 8.5. Integrins Inhibitors

Integrins are a major class of cell adhesion receptors for extracellular matrix molecules. They are heavily involved in retinal development, as well as being major regulatory factors in cell adhesion, migration, proliferation, invasion and apoptosis [97,98]. These features make the molecules promising targets for several retinal diseases. Many integrin inhibitor molecules are currently under investigation, including Risuteganib (Luminate, Allegro Ophthalmics, CA, USA), THR-687 (Oxurion, Leuven, Belgium), SF-0166 (SciFluor Life Sciences, Boston, MA, USA), Volociximab (Ophthotech Corporation, New York, NY, USA, Now Iveric Bio, New York, NY, USA) and JSM-6427 (Takeda Pharmaceutical Company, Tokyo, Japan) [98].

### 8.6. KSI-301

KSI-301 (KODIAK sciences, Palo Alto, CA, USA), is a new generation antibody biopolymer conjugate resulting from the combination of humanized anti-VEGF monoclonal antibody and a phosphorylcholine-based polymer, which has been developed to increase the duration of anti-VEGF action. This molecule is under investigation in a phase 1b trial (NCT03790852), showing promising results in neovascular age-related macular degeneration, diabetic macular edema and retinal vein occlusion, and in a DAZZLE Phase 2 trial (NCT04049266) involving only neovascular age-related macular degeneration patients.

### 8.7. OPT-302

OPT-302 is a VEGF-C/D inhibitor, under investigation in a Phase 2b clinical trial (NCT03345082).

## 9. Tyrosine Kinase Inhibitors

Tyrosine kinase inhibitors are important factors involved in the pathogenesis of exudative retinal diseases, and act as promoters of hypoxia-induced VEGF-A and PDGF molecular cascades [99]. PAN-90806 (PanOptica, Mount Arlington, NJ, USA) is a topical drop formulation of is a tyrosine kinase inhibitor, under investigation in a Phase 1/2 clinical trial (NCT03479372). Sunitinib maleate is an intravitreal tyrosine kinase inhibitor formulation [100], under investigation through ADAGIO Phase 1/2a (NCT03249740) and ALTISSIMO Phase 2b (NCT03953079) clinical trials.

### Squalamine

Squalamine is an aminosterol antibiotic extracted from the shark Squalus acanthias and is known to have antiangiogenic and antitumoral properties [101]. Animal models have provided evidence of improvements in the stages of retinopathy and in neovascularizations [102,103]. Squalamine is also available in topical eyedrop form, to be administered alone or in combination with intravitreal anti-VEGF, providing promising preliminary results [104].

## 10. Retinal Drugs for Non-Exudative Diseases

Unlike exudative retinal diseases, non-exudative retinal diseases lack any approved treatment, to date. These disorders, caused by multifactorial etiopathogenesis, including environmental and genetic factors, mainly involve the progressive degeneration of retinal cells, with onset of retinal atrophy. Most non-exudative retinal diseases start with the degeneration of the retinal pigment epithelium-photoreceptor complex; outer retinal impairment and atrophy is followed by damage to the inner retinal cells, with irremediable loss of retinal function [105,106,107].

Treatment for non-exudative retinal diseases is based on nutraceutics, which employs a combination of different natural substances known to slow down degenerative processes. Although several molecules are under investigation, most of the evidence concerns formulations developed for dry age-related macular degeneration by Age-Related Eye Disease Studies 1 and 2 (AREDS1 and AREDS2) [108]. The AREDS1 formulation contained vitamin C (500 mg), vitamin E (273 mg/473 IU), beta-carotene (15 mg), zinc (80 mg), and copper (2 mg) [109], whereas the AREDS2 formulation differed in the removal of beta-carotene and addition of lutein/zeaxanthin and omega-3 long-chain polyunsaturated fatty acid [110]. Vitamins and ions are important antioxidants and promote enzymatic functions. Lutein/zeaxanthin produce many effects, including removing reactive oxygen species and protecting against photooxidative stress. Omega-3 long-chain polyunsaturated fatty acids are key cell membrane components and are involved in many metabolic pathways. These nutraceutical supplements have been associated with a slowing in the progression of geographic atrophy and a reduced probability of experiencing advances in the stages of age-related macular degeneration. Different formulations of vitamins, polyunsaturated fatty acids, minerals and other compounds have been proposed for inherited retinal dystrophies, supported by modest evidence regarding their clinical efficacy [111].

In this section, we provide a brief survey of the most promising molecules under investigation.

### 10.1. Complement Inhibitors

Complement inhibitors [112] have already been discussed as a potential new treatment for exudative retinal diseases. In the context of dry retinal diseases, their role should cover the inhibition of the molecular mechanisms leading to cell degeneration and apoptosis. Current complement inhibitor drugs under investigation include Lampalizumab (NCT01229215), Zimura (ARC-1905) (NCT02686658), APL-4 (POT-4/AL-78898A) (NCT03525613-NCT03525600), CLG561 (NCT02515942) and LFG316 (NCT01527500).

### 10.2. Brimonidine

Brimonidine is a selective α2 adrenergic (α2A) receptor agonist used as intraocular pressure-lowering medication in glaucoma. Previous evidence suggested this molecule plays a neuroprotective role through as yet poorly understood mechanisms [113,114]. This molecule is under investigation in a Brimonidine Intravitreal Implant in Geographic Atrophy Secondary to Age-related Macular Degeneration (BEACON) phase 2 study (NCT02087085).

## 11. Integrin Inhibitors

The rationale for the use of integrin inhibitors in dry retinal diseases is similar to those described above for exudative diseases. Risuteganib (Alg-1001) (NCT03626636) is currently under investigation for non-exudative age-related macular degeneration. This molecule has been found to be associated with reduced oxidative stress and improved retinal homeostasis.

### H+/K+ ATPase Proton Pump Inhibitors

Soraprazan reversibly binds proton pump hydrogen-potassium adenosine triphosphatase (H+/K+ ATPase) and was originally developed to treat gastro-esophageal reflux disease. Recent evidence suggests Soraprazan can help remove retinal lipofuscin accumulations, slowing the onset and progression of outer retinal atrophy. Soraprazan is currently under investigation in Stargardt retinal dystrophy [115].

## 12. Conclusions

This review has provided a general rundown of the state of the art regarding pharmacological treatment for exudative and dry retinal diseases and a summary of intriguing new prospects. Retinal diseases represent the final step in an extremely complex cascade of events involving several mediators and molecules. While the clinical course of exudative retinal diseases changed radically after the introduction of intravitreal drugs, the management of dry retinal diseases is still short of therapeutic bullets. The molecules under investigation for both kinds of diseases offer some very interesting prospects, as a result of a better understanding of the pathogenic mechanisms leading to the onset and progression of retinal impairment. These molecules focus their activity on a wider range of therapeutic targets. With this in mind, future studies should concentrate more closely on multitarget approaches and on treatments of longer duration, as they represent current challenges and the best chance of achieving important breakthroughs in improving the outcome of retinal diseases, not to mention optimizing the use of public health system resources.

## Data Availability

Data are available after a formal request to the corresponding author.

## References

[1] Díaz-Coránguez M., Ramos C., Antonetti D.A. (2017). The inner blood-retinal barrier: Cellular basis and development. Vision Res..

[2] Antonetti D.A., Klein R., Gardner T.W. (2012). Diabetic retinopathy. N. Engl. J. Med..

[3] De Jong P.T. (2006). Age-related macular degeneration. N. Engl. J. Med..

[4] Davoudi S., Papavasileiou E., Roohipoor R., Cho H., Kudrimoti S., Hancock H., Hoadley S., Andreoli C., Husain D., James M. (2016). Optical coherence tomography characteristics of macular edema and hard exudates and their association with lipid serum levels in type 2 diabetes. Retina.

[5] Jager R.D., Mieler W.F., Miller J.W. (2008). Age-related macular degeneration. N. Engl. J. Med..

[6] Jost B.F., Alexander M.F., Maguire M.G., Fine S.L., Chamberlin J.A., Murphy R.P. (1988). Laser treatment for choroidal neovascularization outside randomized clinical trials. Arch. Ophthalmol..

[7] Shah C.P., Chen C. (2011). Review of therapeutic advances in diabetic retinopathy. Ther. Adv. Endocrinol. Metab..

[8] Stenner A.M., Frederiksen K.H., Grauslund J. (2020). Is there still a role of macular laser treatment in branch retinal vein occlusion in the era of intravitreal injections?. Acta Ophthalmol..

[9] Ferrara N., Carver-Moore K., Chen H., Dowd M., Lu L., O’Shea K.S., Powell-Braxton L., Hillan K.J., Moore M.W. (1996). Heterozygous embryonic lethality induced by targeted inactivation of the VEGF gene. Nature.

[10] Breier G. (2000). Functions of the VEGF/VEGF receptor system in the vascular system. Semin. Thromb. Hemost..

[11] Takahashi H., Shibuya M. (2005). The vascular endothelial growth factor (VEGF)/VEGF receptor system and its role under physiological and pathological conditions. Clin. Sci..

[12] Yancopoulos G.D., Davis S., Gale N.W., Rudge J.S., Wiegand S.J., Holash J. (2000). Vascular-specific growth factors and blood vessel formation. Nature.

[13] Jussila L., Alitalo K. (2000). Vascular growth factors and lymphangiogenesis. Physiol. Rev..

[14] Wang G.L., Semenza G.L. (1995). Purification and characterization of hypoxia-inducible factor 1. J. Biol. Chem..

[15] Grant M.B., Mames R.N., Fitzgerald C., Ellis E.A., Caballero S., Chegini N., Guy J. (1993). Insulin-like growth factor I as an angiogenic agent. In vivo and in vitro studies. Ann. N. Y. Acad. Sci..

[16] Miller J.W., Adamis A.P., Aiello L.P. (1997). Vascular endothelial growth factor in ocular neovascularization and proliferative diabetic retinopathy. Diabetes Metab. Rev..

[17] Stone J., Itin A., Alon T., Pe’Er J., Gnessin H., Chan-Ling T., Keshet E. (1995). Development of retinal vasculature is mediated by hypoxia-induced vascular endothelial growth factor (VEGF) expression by neuroglia. J. Neurosci..

[18] Reichenbach A., Bringmann A. (2020). Glia of the human retina. Glia.

[19] Ida H., Tobe T., Nambu H., Matsumura M., Uyama M., Campochiaro P.A. (2003). RPE cells modulate subretinal neovascularization, but do not cause regression in mice with sustained expression of VEGF. Investig. Ophthalmol. Vis. Sci..

[20] Caldwell R.B., Bartoli M., Behzadian M.A., El-Remessy A.E., Al-Shabrawey M., Platt D.H., Liou G.I., Caldwell R.W. (2005). Vascular endothelial growth factor and diabetic retinopathy: Role of oxidative stress. Curr. Drug Targets.

[21] Shankar A., Mitchell P., Rochtchina E., Tan J., Wang J.J. (2007). Association between circulating white blood cell count and long-term incidence of age-related macular degeneration: The Blue Mountains Eye Study. Am. J. Epidemiol..

[22] Watanabe D., Suzuma K., Matsui S., Kurimoto M., Kiryu J., Kita M., Suzuma I., Ohashi H., Ojima T., Murakami T. (2005). Erythropoietin as a retinal angiogenic factor in proliferative diabetic retinopathy. N. Engl. J. Med..

[23] Betsholtz C. (2004). Insight into the physiological functions of PDGF through genetic studies in mice. Cytokine Growth Factor Rev..

[24] Hageman G.S., Luthert P.J., Victor Chong N.H., Johnson L.V., Anderson D.H., Mullins R.F. (2001). An integrated hypothesis that considers drusen as biomarkers of immune-mediated processes at the RPE-Bruch’s membrane interface in aging and age-related macular degeneration. Prog. Retin. Eye Res..

[25] Zhang S.X., Wang J.J., Gao G., Parke K., Ma J.X. (2006). Pigment epithelium-derived factor downregulates vascular endothelial growth factor (VEGF) expression and inhibits VEGF-VEGF receptor 2 binding in diabetic retinopathy. J. Mol. Endocrinol..

[26] Kociok N., Joussen A.M. (2007). Varied expression of functionally important genes of RPE and choroid in the macula and in the periphery of normal human eyes. Graefes Arch. Clin. Exp. Ophthalmol..

[27] Yang J.C., Haworth L., Sherry R.M., Hwu P., Schwartzentruber D.J., Topalian S.L., Steinberg S.M., Chen H.X., Rosenberg S.A. (2003). A randomized trial of bevacizumab, an anti-vascular endothelial growth factor antibody, for metastatic renal cancer. N. Engl. J. Med..

[28] Rosenfeld P.J., Moshfeghi A.A., Puliafito C.A. (2005). Optical coherence tomography findings after an intravitreal injection of bevacizumab (avastin) for neovascular age-related macular degeneration. Ophthalm. Surg. Lasers Imag..

[29] Kreisl T.N., Kim L., Moore K., Duic P., Royce C., Stroud I., Garren N., Mackey M., Butman J.A., Camphausen K. (2009). Phase II trial of single-agent bevacizumab followed by bevacizumab plus irinotecan at tumor progression in recurrent glioblastoma. J. Clin. Oncol..

[30] Spasic M., Chow F., Tu C., Nagasawa D.T., Yang I. (2012). Molecular characteristics and pathways of Avastin for the treatment of glioblastoma multiforme. Neurosurg. Clin. N. Am..

[31] Stefanini F.R., Arevalo J.F., Maia M. (2013). Bevacizumab for the management of diabetic macular edema. World J. Diabetes.

[32] Avery R.L., Pieramici D.J., Rabena M.D., Castellarin A.A., Nasir M.A., Giust M.J. (2006). Intravitreal bevacizumab (Avastin) for neovascular age-related macular degeneration. Ophthalmology.

[33] Balla S., Zold E., Potor L., Lukucz B., Vajas A., Ujhelyi B., Nagy V. (2020). Analysis of intravitreal bevacizumab treatment for macular oedema due to retinal vein occlusion. Eur. J. Ophthalmol..

[34] Lee J.H., Canny M.D., De Erkenez A., Krilleke D., Ng Y.S., Shima D.T., Pardi A., Jucker F. (2005). A therapeutic aptamer inhibits angiogenesis by specifically targeting the heparin binding domain of VEGF165. Proc. Natl. Acad. Sci. USA.

[35] Gragoudas E.S., Adamis A.P., Cunningham ETJr Feinsod M., Guyer D.R. (2004). VEGF Inhibition Study in Ocular Neovascularization Clinical Trial Group. Pegaptanib for neovascular age-related macular degeneration. N. Engl. J. Med..

[36] Ferrara N., Damico L., Shams N., Lowman H., Kim R. (2006). Development of ranibizumab, an anti-vascular endothelial growth factor antigen binding fragment, as therapy for neovascular age-related macular degeneration. Retina.

[37] Lowe J., Araujo J., Yang J., Reich M., Oldendorp A., Shiu V., Quarmby V., Lowman H., Lien S., Gaudreault J. (2007). Ranibizumab inhibits multiple forms of biologically active vascular endothelial growth factor in vitro and in vivo. Exp. Eye Res..

[38] Vaidyanathan U., Moshirfar M. (2021). Ranibizumab. 2020. StatPearls.

[39] Platania C.B., Di Paola L., Leggio G.M., Romano G.L., Drago F., Salomone S., Bucolo C. (2015). Molecular features of interaction between VEGFA and anti-angiogenic drugs used in retinal diseases: A computational approach. Front. Pharmacol..

[40] Yang J., Wang X., Fuh G., Yu L., Wakshull E., Khosraviani M., Day E.S., Demeule B., Liu J., Shire S.J. (2014). Comparison of binding characteristics and in vitro activities of three inhibitors of vascular endothelial growth factor *A*. Mol. Pharm..

[41] Holash J., Davis S., Papadopoulos N., Croll S.D., Ho L., Russell M., Boland P., Leidich R., Hylton D., Burova E. (2002). VEGF-Trap: A VEGF blocker with potent antitumor effects. Proc. Natl. Acad. Sci. USA.

[42] Cheng Y.D., Yang H., Chen G.Q., Zhang Z.C. (2013). Molecularly targeted drugs for metastatic colorectal cancer. Drug Des. Devel. Ther..

[43] Mansour A.M., Al-Ghadban S.I., Yunis M.H., El-Sabban M.E. (2015). Ziv-aflibercept in macular disease. Br. J. Ophthalmol..

[44] Zhang M., Zhang J., Yan M., Li H., Yang C., Yu D. (2008). Recombinant anti-vascular endothelial growth factor fusion protein efficiently suppresses choridal neovascularization in monkeys. Mol. Vis..

[45] Suto K., Yamazaki Y., Morita T., Mizuno H. (2005). Crystal structures of novel vascular endothelial growth factors (VEGF) from snake venoms: Insight into selective VEGF binding to kinase insert domain-containing receptor but not to fms-like tyrosine kinase-1. J. Biol. Chem..

[46] Yannuzzi N.A., Freund K.B. (2019). Brolucizumab: Evidence to date in the treatment of neovascular age-related macular degeneration. Clin. Ophthalmol..

[47] Sharma A., Kumar N., Kuppermann B.D., Bandello F. (2020). Brolucizimab-leading an era of structural revolution for long-term VEGF suppression. Eye.

[48] Nguyen Q.D., Das A., Do D.V., Dugel P.U., Gomes A., Holz F.G., Koh A., Pan C.K., Sepah Y.J., Patel N. (2020). Brolucizumab: Evolution through Preclinical and Clinical Studies and the Implications for the Management of Neovascular Age-Related Macular Degeneration. Ophthalmology.

[49] Tadayoni R., Sararols L., Weissgerber G., Verma R., Clemens A., Holz F.G. (2021). Brolucizumab: A Newly Developed Anti-VEGF Molecule for the Treatment of Neovascular Age-Related Macular Degeneration. Ophthalmologica.

[50] Stumpp M.T., Binz H.K., Amstutz P. (2008). DARPins: A new generation of protein therapeutics. Drug Discov. Today.

[51] Souied E.H., Devin F., Mauget-Faÿsse M., Kolář P., Wolf-Schnurrbusch U., Framme C., Gaucher D., Querques G., Stumpp M.T., Wolf S. (2014). MP0112 Study Group. Treatment of exudative age-related macular degeneration with a designed ankyrin repeat protein that binds vascular endothelial growth factor: A phase I/II study. Am. J. Ophthalmol..

[52] Rodrigues G.A., Mason M., Christie L.A., Hansen C., Hernandez L.M., Burke J., Luhrs K.A., Hohman T.C. (2018). Functional Characterization of Abicipar-Pegol, an Anti-VEGF DARPin Therapeutic That Potently Inhibits Angiogenesis and Vascular Permeability. Invest. Ophthalmol. Vis. Sci..

[53] Nicolò M., Ferro Desideri L., Vagge A., Traverso C.E. (2021). Faricimab: An investigational agent targeting the Tie-2/angiopoietin pathway and VEGF-A for the treatment of retinal diseases. Exp. Opin. Investig. Drugs.

[54] Solomon S.D., Lindsley K., Vedula S.S., Krzystolik M.G., Hawkins B.S. (2014). Anti-vascular endothelial growth factor for neovascular age-related macular degeneration. Cochrane Database Syst. Rev..

[55] Virgili G., Parravano M., Evans J.R., Gordon I., Lucenteforte E. (2018). Anti-vascular endothelial growth factor for diabetic macular oedema: A network meta-analysis. Cochrane Database Syst. Rev..

[56] Zhu Y., Zhang T., Xu G., Peng L. (2016). Anti-vascular endothelial growth factor for choroidal neovascularisation in people with pathological myopia. Cochrane Database Syst. Rev..

[57] Braithwaite T., Nanji A.A., Lindsley K., Greenberg P.B. (2014). Anti-vascular endothelial growth factor for macular oedema secondary to central retinal vein occlusion. Cochrane Database Syst. Rev..

[58] Shalchi Z., Mahroo O., Bunce C., Mitry D. (2020). Anti-vascular endothelial growth factor for macular oedema secondary to branch retinal vein occlusion. Cochrane Database Syst. Rev..

[59] Dugel P.U., Koh A., Ogura Y., Jaffe G.J., Schmidt-Erfurth U., Brown D.M., Gomes A.V., Warburton J., Weichselberger A., Holz F.G. (2020). HAWK and HARRIER Study Investigators. HAWK and HARRIER: Phase 3, Multicenter, Randomized, Double-Masked Trials of Brolucizumab for Neovascular Age-Related Macular Degeneration. Ophthalmology.

[60] Clinical Trials.gov A Study to Evaluate the Efficacy and Safety of Faricimab in Participants with Neovascular Age-Related Macular Degeneration (TENAYA). https://clinicaltrials.gov/ct2/show/NCT03823287.

[61] Clinical Trials.gov A Study to Evaluate the Efficacy and Safety of Faricimab in Participants with Neovascular Age-Related Macular Degeneration (LUCERNE). https://clinicaltrials.gov/ct2/show/NCT03823300.

[62] Xu H., Chen M., Forrester J.V. (2009). Para-inflammation in the aging retina. Prog. Retin. Eye Res..

[63] Tang J., Kern T.S. (2011). Inflammation in diabetic retinopathy. Prog. Retin. Eye Res..

[64] Joussen A.M., Murata T., Tsujikawa A., Kirchhof B., Bursell S.E., Adamis A.P. (2001). Leukocyte-mediated endothelial cell injury and death in the diabetic retina. Am. J. Pathol..

[65] Koss M.J., Pfister M., Rothweiler F., Michaelis M., Cinatl J., Schubert R., Koch F.H. (2012). Comparison of cytokine levels from undiluted vitreous of untreated patients with retinal vein occlusion. Acta Ophthalmol..

[66] De Smet M.D., Taylor S.R., Bodaghi B., Miserocchi E., Murray P.I., Pleyer U., Zierhut M., Barisani-Asenbauer T., LeHoang P., Lightman S. (2011). Understanding uveitis: The impact of research on visual outcomes. Prog. Retin. Eye Res..

[67] Olivares-González L., Velasco S., Campillo I., Rodrigo R. (2021). Retinal Inflammation, Cell Death and Inherited Retinal Dystrophies. Int. J. Mol. Sci..

[68] National Center for Biotechnology Information 2021. PubChem Compound Summary for CID 6436, Triamcinolone Acetonide. https://pubchem.ncbi.nlm.nih.gov/compound/Triamcinolone-acetonide.

[69] Mansoor S., Kuppermann B.D., Kenney M.C. (2009). Intraocular sustained-release delivery systems for triamcinolone acetonide. Pharm. Res..

[70] Mizuno S., Nishiwaki A., Morita H., Miyake T., Ogura Y. (2007). Effects of periocular administration of triamcinolone acetonide on leukocyte-endothelium interactions in the ischemic retina. Investig. Ophthalmol. Vis. Sci..

[71] Spandau U.H., Sauder G., Schubert U., Hammes H.P., Jonas J.B. (2005). Effect of triamcinolone acetonide on proliferation of retinal endothelial cells in vitro and in vivo. Br. J. Ophthalmol..

[72] Narayanan R., Mungcal J.K., Kenney M.C., Seigel G.M., Kuppermann B.D. (2006). Toxicity of triamcinolone acetonide on retinal neurosensory and pigment epithelial cells. Invest. Ophthalmol. Vis. Sci..

[73] National Center for Biotechnology Information 2021. PubChem Compound Summary for CID 5743, Dexamethasone. https://pubchem.ncbi.nlm.nih.gov/compound/Dexamethasone.

[74] Chang-Lin J.E., Attar M., Acheampong A.A., Robinson M.R., Whitcup S.M., Kuppermann B.D., Welty D. (2011). Pharmacokinetics and pharmacodynamics of a sustained-release dexamethasone intravitreal implant. Invest. Ophthalmol. Vis. Sci..

[75] Fallico M., Maugeri A., Lotery A., Longo A., Bonfiglio V., Russo A., Avitabile T., Furino C., Cennamo G., Barchitta M. (2021). Fluocinolone acetonide vitreous insert for chronic diabetic macular oedema: A systematic review with meta-analysis of real-world experience. Sci. Rep..

[76] National Center for Biotechnology Information 2021. PubChem Compound Summary for CID 6215, Fluocinolone Acetonide. https://pubchem.ncbi.nlm.nih.gov/compound/Fluocinolone-acetonide.

[77] Campochiaro P.A., Nguyen Q.D., Hafiz G., Bloom S., Brown D.M., Busquets M., Ciulla T., Feiner L., Sabates N., Billman K. (2013). FAMOUS Study Group. Aqueous levels of fluocinolone acetonide after administration of fluocinolone acetonide inserts or fluocinolone acetonide implants. Ophthalmology.

[78] Kane F.E., Green K.E. (2015). Ocular pharmacokinetics of fluocinolone acetonide following Iluvien implantation in the vitreous humor of rabbits. J. Ocul. Pharmacol. Ther..

[79] Jaffe G.J., Pavesio C.E. (2020). Study Investigators. Effect of a Fluocinolone Acetonide Insert on Recurrence Rates in Noninfectious Intermediate, Posterior, or Panuveitis: Three-Year Results. Ophthalmology.

[80] Rittiphairoj T., Mir T.A., Li T., Virgili G. (2020). Intravitreal steroids for macular edema in diabetes. Cochrane Database Syst. Rev..

[81] Zhang Y., Duan J., Chang T., Li X., Wang M., Zhang M. (2020). Comparative efficacy of intravitreal pharmacotherapy for macular edema secondary to retinal vein occlusion: A protocol for the systematic review and network meta-analysis. Medicine.

[82] Brady C.J., Villanti A.C., Law H.A., Rahimy E., Reddy R., Sieving P.C., Garg S.J., Tang J. (2016). Corticosteroid implants for chronic non-infectious uveitis. Cochrane Database Syst. Rev..

[83] James E.R. (2007). The etiology of steroid cataract. J. Ocul. Pharmacol. Ther..

[84] Johnson D., Gottanka J., Flügel C., Hoffmann F., Futa R., Lütjen-Drecoll E. (1997). Ultrastructural changes in the trabecular meshwork of human eyes treated with corticosteroids. Arch. Ophthalmol..

[85] Yemanyi F., Baidouri H., Burns A.R., Raghunathan V. (2020). Dexamethasone and Glucocorticoid-Induced Matrix Temporally Modulate Key Integrins, Caveolins, Contractility, and Stiffness in Human Trabecular Meshwork Cells. Invest. Ophthalmol. Vis. Sci..

[86] Kersey J.P., Broadway D.C. (2006). Corticosteroid-induced glaucoma: A review of the literature. Eye.

[87] Sharma N.K., Prabhakar S., Gupta A., Singh R., Gupta P.K., Gupta P.K., Anand A. (2012). New biomarker for neovascular age-related macular degeneration: Eotaxin-2. DNA Cell Biol..

[88] Grisanti S., Canbek S., Kaiserling E., Adam A., Lafaut B., Gelisken F., Szurman P., Henke-Fahle S., Oficjalska-Mlynczak J., Bartz-Schmidt K.U. (2004). Expression of endoglin in choroidal neovascularization. Exp. Eye Res..

[89] Shen W., Lee S.R., Yam M., Zhu L., Zhang T., Pye V., Mathai A.E., Shibagaki K., Zhang J.Z., Matsugi T. (2018). A Combination Therapy Targeting Endoglin and VEGF-A Prevents Subretinal Fibro-Neovascularization Caused by Induced Müller Cell Disruption. Investig. Ophthalmol. Vis. Sci..

[90] Frederick P.A., Kleinman M.E. (2014). The Immune System and AMD. Curr. Ophthalmol. Rep..

[91] Pan W.W., Lin F., Fort P.E. (2021). The innate immune system in diabetic retinopathy. Prog. Retin. Eye Res..

[92] Sodi A., Passerini I., Bacherini D., Boni L., Palchetti S., Murro V., Caporossi O., Mucciolo D.P., Franco F., Vannozzi L. (2018). CFH Y402H polymorphism in Italian patients with age-related macular degeneration, retinitis pigmentosa, and Stargardt disease. Ophthalm. Genet..

[93] Williams M.A., McKay G.J., Chakravarthy U. (2014). Complement inhibitors for age-related macular degeneration. Cochrane Database Syst. Rev..

[94] Park D.H., Connor K.M., Lambris J.D. (2019). The Challenges and Promise of Complement Therapeutics for Ocular Diseases. Front. Immunol..

[95] Wu J., Sun X. (2019). Complement system and age-related macular degeneration: Drugs and challenges. Drug Des. Devel. Ther..

[96] Gonzales C.R., Burian B. (2017). A Phase 2 Study (EMERGE) Evaluating Repeated Intravitreal Administration of ICON-1 in Patients with Choroidal Neovascularization (CNV) Secondary to Age-related Macular Degeneration (AMD). Investig. Ophthalmol. Vis. Sci..

[97] Li M., Sakaguchi D.S. (2004). Inhibition of integrin-mediated adhesion and signaling disrupts retinal development. Dev. Biol..

[98] Bhatwadekar A.D., Kansara V., Luo Q., Ciulla T. (2020). Anti-integrin therapy for retinovascular diseases. Exp. Opin. Investig. Drugs.

[99] Hwang S., Seong H., Ryu J., Jeong J.Y., Kang T.S., Nam K.Y., Seo S.W., Kim S.J., Kang S.S., Han Y.S. (2020). Phosphorylation of STAT3 and ERBB2 mediates hypoxia-induced VEGF release in ARPE-19 cells. Mol. Med. Rep..

[100] Tsujinaka H., Fu J., Shen J., Yu Y., Hafiz Z., Kays J., McKenzie D., Cardona D., Culp D., Peterson W. (2020). Sustained treatment of retinal vascular diseases with self-aggregating sunitinib microparticles. Nat. Commun..

[101] Sills A.K., Williams J.I., Tyler B.M., Epstein D.S., Sipos E.P., Davis J.D., McLane M.P., Pitchford S., Cheshire K., Gannon F.H. (1998). Squalamine inhibits angiogenesis and solid tumor growth in vivo and perturbs embryonic vasculature. Cancer Res..

[102] Higgins R.D., Sanders R.J., Yan Y., Zasloff M., Williams J.I. (2000). Squalamine improves retinal neovascularization. Investig. Ophthalmol. Vis. Sci..

[103] Higgins R.D., Yan Y., Geng Y., Zasloff M., Williams J.I. (2004). Regression of retinopathy by squalamine in a mouse model. Pediatr. Res..

[104] Wroblewski J.J., Hu A.Y. (2016). Topical Squalamine 0.2% and Intravitreal Ranibizumab 0.5 mg as Combination Therapy for Macular Edema Due to Branch and Central Retinal Vein Occlusion: An Open-Label, Randomized Study. Ophthalm. Surg. Lasers Imag. Retina.

[105] Boyer D.S., Schmidt-Erfurth U., van Lookeren Campagne M., Henry E.C., Brittain C. (2017). The pathophysiology of geographic atrophy secondary to age-related macular degeneration and the complement pathway as a therapeutic target. Retina.

[106] Boon C.J., den Hollander A.I., Hoyng C.B., Cremers F.P., Klevering B.J., Keunen J.E. (2008). The spectrum of retinal dystrophies caused by mutations in the peripherin/RDS gene. Prog. Retin. Eye Res..

[107] Bird A.C. (1995). Retinal photoreceptor dystrophies. Am. J. Ophthalmol..

[108] Parodi M.B., Zucchiatti I., Cicinelli M.V., Cascavilla M.L., Bandello F. (2016). Nutritional supplementation in age-related macular degeneration. Retina.

[109] Age-Related Eye Disease Study Research Group (2001). A randomized, placebo-controlled, clinical trial of high-dose supplementation with vitamins C and E, beta carotene, and zinc for age-related macular degeneration and vision loss: AREDS report no. 8. Arch. Ophthalmol..

[110] Group A.R., Chew E.Y., Clemons T., SanGiovanni J.P., Danis R., Domalpally A., McBee W., Sperduto R., Ferris F.L. (2012). The Age-Related Eye Disease Study 2 (AREDS2): Study design and baseline characteristics (AREDS2 report number 1). Ophthalmology.

[111] Brito-García N., Del Pino-Sedeño T., Trujillo-Martín M.M., Coco R.M., Rodríguez de la Rúa E., Del Cura-González I., Serrano-Aguilar P. (2017). Effectiveness and safety of nutritional supplements in the treatment of hereditary retinal dystrophies: A systematic review. Eye.

[112] Liao D.S., Grossi F.V., El Mehdi D., Gerber M.R., Brown D.M., Heier J.S., Wykoff C.C., Singerman L.J., Abraham P., Grassmann F. (2020). Complement C3 Inhibitor Pegcetacoplan for Geographic Atrophy Secondary to Age-Related Macular Degeneration: A Randomized Phase 2 Trial. Ophthalmology.

[113] Saylor M., McLoon L.K., Harrison A.R., Lee M.S. (2009). Experimental and clinical evidence for brimonidine as an optic nerve and retinal neuroprotective agent: An evidence-based review. Arch. Ophthalmol..

[114] Nizari S., Guo L., Davis B.M., Normando E.M., Galvao J., Turner L.A., Bizrah M., Dehabadi M., Tian K., Cordeiro M.F. (2016). Non-amyloidogenic effects of α2 adrenergic agonists: Implications for brimonidine-mediated neuroprotection. Cell Death Dis..

[115] Hoyng C.C.B., Lotery A., Stingl K., Boon C., Parodi M., Dhooge P., Peters T., Klein W., Fsadni M.G., Müller H. (2019). Designing a clinical trial to evaluate the safety and efficacy of oral soraprazan in Stargardt Disease. Investig. Ophthalmol. Vis. Sci..

